# Evaluation of cerebral hemodynamic status in patients with unilateral symptomatic carotid artery stenosis during motor tasks, through use of transcranial Doppler sonography

**DOI:** 10.1590/0004-282X-ANP-2020-0571

**Published:** 2022-01-10

**Authors:** Aysel MILANLIOGLU, Aslı YAMAN, Mehmet KOLUKISA, Talip ASIL

**Affiliations:** 1 Yüzüncü Yıl University, Faculty of Medicine, Department of Neurology, Van, Turkey.; 2 Bezmialem Vakıf University, Faculty of Medicine, Department of Neurology, İstanbul, Turkey.

**Keywords:** Carotid Stenosis, Vasomotor System, Ultrasonography, Doppler, Transcranial, Estenose das Carótidas, Sistema Vasomotor, Ultrassonografia Doppler Transcraniana

## Abstract

**Background:** Carotid artery stenosis increases cerebral ischemic event risk through changing different cerebral hemodynamic parameters. **Objective:** To investigate how cerebral hemodynamics in the M1 segment of middle cerebral artery change in patients with carotid artery stenosis, after motor tasks using transcranial Doppler sonography (TCD). **Methods:** Thirty-two healthy subjects and 30 patients with unilateral symptomatic carotid artery stenosis were recruited. The patient population was divided into three groups according to the degree of stenosis (group 1: ≥50 to 69%, group 2: 70 to 89% and group 3: ≥90 to 99%). TCD was used to measure the pulsatility index (PI) and cerebral vasomotor reactivity (CVR). **Results:** In the patient group, significant differences for symptomatic side PI values (p=0.01) and mean CVR increases (p=0.05) were observed, compared with the healthy controls. However, the difference was not statistically significant for asymptomatic side PI values and mean CVR increases. The results from the intergroup comparison showed significantly higher percentages of symptomatic and asymptomatic side CVR increases in group 1, compared with groups 2 and 3 (p=0.001 and p=0.002, respectively). **Conclusions:** Our study showed that cerebral autoregulation and hemodynamic mechanisms are impaired in patients with carotid artery stenosis. Furthermore, the impairment of PI and CVR tends to get worse with increasing degrees of stenosis. In addition, this study demonstrated that assessment of these two hemodynamic parameters in clinical practice might be helpful for monitoring the progress of carotid artery stenosis.

## RESUMO

**Antecedentes:** A estenose da artéria carótida aumenta o risco de evento isquêmico cerebral por meio da alteração de diferentes parâmetros hemodinâmicos cerebrais. **Objetivo:** Investigar como a hemodinâmica cerebral no segmento M1 da artéria cerebral média se altera em pacientes com estenose da artéria carótida, após tarefas motoras com ultrassonografia Doppler transcraniana (DTC). **Métodos:** Foram recrutados trinta e dois indivíduos saudáveis e 30 pacientes com estenose da artéria carótida sintomática unilateral. A população de pacientes foi dividida em três grupos de acordo com o grau de estenose (grupo 1: ≥50 a 69%, grupo 2: 70 a 89% e grupo 3: ≥90 a 99%). A DTC foi usada para medir o índice de pulsatilidade (IP) e a reatividade vasomotora cerebral (RVC). **Resultados:** No grupo de pacientes, foram observadas diferenças significativas para os valores de IP do lado sintomático (p=0,01) e aumentos médios da RVC (p=0,05), em comparação com os controles saudáveis. No entanto, a diferença não foi estatisticamente significativa para os valores de IP laterais assintomáticos e aumentos médios de RVC. Os resultados da comparação intergrupos mostraram percentagens significativamente maiores de aumentos da RVC do lado sintomático e assintomático no grupo 1, em comparação com os grupos 2 e 3 (p=0,001 e p=0,002, respectivamente). **Conclusões:** Nosso estudo mostrou que a autorregulação cerebral e os mecanismos hemodinâmicos estão prejudicados em pacientes com estenose da artéria carótida. Além disso, o comprometimento do IP e da RVC tende a piorar com o aumento dos graus de estenose. Além disso, este estudo demonstrou que a avaliação desses dois parâmetros hemodinâmicos na prática clínica pode ser útil para monitorar a evolução da estenose da artéria carótida.

## INTRODUCTION

The relationship between internal carotid artery stenosis and stroke has been widely examined in the medical literature. This disease is reported in approximately 15–20% of stroke patients^1^, and older age definitely increases the risk. It has been reported that 7% of women and 9% of men have more than 50% carotid artery stenosis above age 75 years^2^.

There are many studies demonstrating that carotid artery stenosis increases the cerebral ischemic event risk through changing different cerebral hemodynamic parameters^3,4^. However, how these cerebral hemodynamic parameters alter in relation to the degree of stenosis in the carotid artery remains unexplored. It would be expected that these two physiological mechanisms affect each other, given that stenosis is likely to cause a reduction in cerebral blood flow and it also decreases the effectiveness of autoregulation^5^. A full understanding of their relationship can aid in clinical practice and in monitoring the progress of stenosis in the carotid artery.

Neurovascular coupling refers to the relationship between local neural activity and subsequent changes in cerebral blood flow. Understanding which aspects of neural activity drive the vascular response is important. Up to now, numerous vascular-based functional brain imaging modalities, like functional magnetic resonance imaging (fMRI), have been used. However, these may not always be feasible, due to their high costs and logistic issues.

Transcranial doppler sonography (TCD) is a relatively cheaper and more accessible method. TCD is a noninvasive technique that detects flow velocities in the cerebral arterioles and hemodynamic changes during specific activation stimuli that are capable of producing changes in cerebral activity and metabolism. This method has been used as an alternative measurement of cerebral blood flow responses to neural activity^6^.

TCD can measure several parameters, including cerebral vasomotor reactivity (CVR). CVR demonstrates the compensatory vasodilatation capacity of cerebral arterioles in response to various specific vasomotor stimuli, such as reduction in systemic blood pressure, changes in oxygen extraction or partial carbon-dioxide pressure, breath-holding, application of vasoactive substances such as acetazolamide, or motor stimulus. Briefly, this parameter gives an idea about individuals’ functional cerebral hemodynamic reserves^7^. Previous studies have indicated that impairment of CVR might be related to various conditions including hypertension^8^, cognitive disorder^9^, diabetes mellitus^10^ and sleep apnea syndrome^11^.

One of the other major factors influencing cerebral hemodynamics is the pulsatility index (PI), which was originally designed to measure vascular resistance. Thus, an increased PI represents probably enhanced cerebrovascular resistance in the cerebral circulation and reduced CVR^12^.

The association between neural activation and enhanced regional cerebral blood flow, due to increased metabolism of the cerebral cortex caused by external stimuli, has now been investigated in many studies^13,14,15,16^. However, none of the previous studies used a motor evoked response as a stimulus. Therefore, we aimed here to monitor changes in blood flow velocity in the middle cerebral artery (MCA) through using TCD in response to a repetitive motor stimulus, in patients with varying degrees of unilateral symptomatic carotid artery stenosis.

## METHODS

### Subjects

Patients with internal carotid artery stenosis of 50 to 99% were selected as participants for the current study. These carotid artery stenosis values were determined through using carotid duplex ultrasound and were measured in accordance with the criteria of the North American Symptomatic Carotid Endarterectomy Trial (NASCET).

Right-handed patients of 18-70 years of age, with unilateral symptomatic internal carotid artery stenosis of ≥50%, and with a history of ischemic stroke or transient ischemic attack (TIA) in the ipsilateral arterial region, were included in this study. Imaging of the patients’ carotid artery was examined by using continuous-wave Doppler and color-flow B mode Doppler ultrasound (Esaote MyLab 30 CV Color Doppler Ultrasound System). In patients with steno-occlusive lesions of ≥70% that were observed via carotid duplex ultrasound, the degree of stenosis was confirmed by means of cervical CT angiography or magnetic resonance angiography.

A detailed medical history, including all major vascular risk factors (hypertension, diabetes mellitus, dyslipidemia and smoking), was established for all participants. Routine blood analyses and neurological examinations were also performed. In addition to antiplatelet drugs, other drugs (insulin, oral antidiabetic treatment, statins and different classes of antihypertensive drugs) were also given as medical treatments to manage vascular risk factors.

Patients with the following were not included as participants in this study: a history of stroke or transient ischemic attack (TIA) during the last 3 months; motor weakness in upper extremities; poor insonation of cranial window; intracranial and/or extra-cranial tandem stenosis; bilateral carotid artery stenosis (greater than 40% shown on the side contralateral to the occlusion); significant alteration in vertebral arteries in a simultaneous vertebra-basilar artery examination; anemia (hematocrit ≤30%) or polycythemia (hematocrit ≥50%) affecting cerebral blood flow; or cognitive disorders. Patients with possible or probable embolizing cardiopathy (atrial fibrillation, mitral valve stenosis, mechanical cardiac valves, recent myocardial infarction, left ventricular thrombus, dilated cardio-myopathies or patent foramen ovale) were also excluded.

Based on these criteria, 30 patients (21 males and 9 females; mean age 67.5 years) and 32 healthy subjects (24 males and 8 females; mean age 65.03 years) were recruited. Moreover, the patient population was divided into three groups: group 1 included 10 patients diagnosed with carotid stenosis of ≥50 to 69% (5 males and 5 females; mean age 69.40±11.34 years); group 2 included 10 patients with stenosis of 70 to 89% (8 males and 2 females; mean age 73.70±5.35 years); and group 3 included 10 patients with stenosis of ≥90 to 99% (8 males and 2 females; mean age 67.50±7.76 years). A population of 32 right-handed, age and gender-matched healthy subjects without carotid stenosis or any neurological abnormality in their history were also enrolled and underwent the same exploration procedures using TCD.

This study was conducted ethically in accordance with the World Medical Association’s Declaration of Helsinki and ethical clearance was obtained from the regional ethics committee. Written and oral informed consent was obtained from the study participants.

### Transcranial doppler sonography procedure

Prior to the TCD procedure, the patients’ systolic and diastolic arterial blood pressures and heart rate were measured following a resting period of 10 minutes in a quiet and calm room. After this process, the patients were asked to lie down comfortably in a supine position. The Sonora TCD system (CareFusion, San Diego, CA, USA) was used for the standard TCD recording protocol. During TCD recording, a 2 MHz pulse doppler transducer probe was placed in a temporal window, and the middle cerebral artery blood flow velocity (MCAv) was measured at depths of between 50 and 60 mm. Systolic blood flow velocity (SBFV), diastolic blood flow velocity (DBFV) and PI were recorded in the M1 segment of the MCA. However, only PI results were analyzed without evaluating SBFV and DBFV separately. Moreover, the Gosling PI formulation was calculated automatically as (systolic velocity-diastolic velocity)/(mean velocity)^9^.

MCAv is a marker of cerebral blood flow in the ipsilateral carotid artery. A baseline recording of MCAv was performed with the subject under baseline conditions (resting and breathing room air). One of the vasodilatory stimuli, a motor task, was then administered in accordance with the motor task protocol mentioned below. MCAv values were separately calculated for each group under the baseline conditions and during the motor task. The relative increase from baseline to motor activation was calculated based on the following equation: [(MCAv activation-MCAv baseline)/(MCAv baseline)]*100 and was expressed as the CVR value. Among the healthy subjects, measurements of PI and CVR were exclusively obtained from the left M1 branch of the MCA. At least three measurements of PI and CVR were performed at a similar depth, and the median value was selected and used in this study.

### Motor task protocol

During the entire procedure, the subjects were tested with closed eyes in a comfortable supine position. For finger movements, the arm was positioned in a guide hinge that allowed movements in only one plane, coinciding with flexion and extension of the fingers. Flexion and extension movements of the fingers were performed every two seconds at the same rate, and this phase was terminated after 30 seconds. The rest condition was 1 minute period preceding each motor task after verification of the absence of oscillation of flow velocity. The subjects were instructed to move fingers in synchrony during the active periods and then to remain in a resting position. The TCD assessment was repeated three times and the mean value was calculated for each subject. Before proceeding to the definitive recording, the subjects were trained to perform the procedure correctly.

All the subjects abstained from drinking alcohol and beverages containing caffeine, and from smoking, for at least 24 hours prior to the examination. All recordings and calculations of CVR and PI were performed in the early morning by the same two operators, who were blinded to clinical and other TCD data.

Statistical analyses were performed by means of a computerized program, the *Statistical Package for the Social Sciences* software (version 13.0). The Pearson’s chi-square test was performed to compare categorical variables among the groups. The age distribution between groups was assessed using Student’s *t* test. The ANOVA test was used to assess the significance of differences among the three subject groups. Correlations between variables were measured and evaluated using Pearson’s correlation coefficient. The statistical significance level was taken to be p≤0.05.

## RESULTS

Among the subject groups, there were no significant differences when the distribution of age, sex and vascular risk factors was taken into consideration. The demographic data and vascular risk factors are presented in Table 1.

**Table 1.** Demographic data and vascular risk factors in carotid stenosis patients and control group.

	Patients (n=30)	Controls (n=32)	p-value
Sex (male/female)	21/9	24/8	NS
Mean age (years)	67.5	65.03	NS
Smoking	6	8	NS
Hypertension	24	20	NS
Hyperlipidemia	27	25	NS
Diabetes mellitus	8	10	NS

NS: non-significant.

The comparison of mean CVR increases and PI values on the symptomatic and asymptomatic sides is summarized in Table2. Although the CVR values were found to be negatively correlated with age and PI values (p<0.01), the PI values were found to be positively correlated with age (p<0.05).

**Table 2.** Comparison of cerebral vasomotor reactivity increase and pulsatility index between carotid stenosis patients and control group.

	Patients		Controls	p-value
CVR increase	Symptomatic side	28.98%	32.37%	0.05
	Asymptomatic side	30.95%		0.40
PI	Symptomatic side	1.25	0.97	0.01
	Asymptomatic side	1.09		0.20

PI: pulsatility index; CVR: cerebral vasomotor reactivity.

From the subgroup analysis, the mean CVR increases on the symptomatic side were 34.78% in group 1, 27.74% in group 2 and 24.44% in group 3. However, the mean CVR increases on the asymptomatic side were 36.09, 30.02 and 26.75%, respectively. The CVR increases on the symptomatic and asymptomatic sides of group 1 were statistically significantly different from those of group 2 (p=0.001) and group 3 (p=0.002). On the other hand, the mean PI values on the symptomatic side were calculated as 0.78 in group 1, 1.43 in group 2 and 1.55 in group 3 while the mean PI values on the asymptomatic side were 0.76, 1.26 and 1.25, respectively. Intergroup comparisons showed that the PI values on the symptomatic and asymptomatic sides in group 1 were statistically significantly lower than those in group 2 (p=0.001) and group 3 (p=0.002).

In addition, the mean CVR increases on the symptomatic side were negatively associated with the degree of stenosis in the carotid artery (p<0.01), while the mean CVR increases on the asymptomatic side were negatively associated with the degree of stenosis in the carotid artery and were positively associated with the mean CVR increases on the symptomatic side (p<0.01). Correlation analysis on the data revealed that the mean PI values on the symptomatic side were negatively correlated with the mean CVR increases on the symptomatic and asymptomatic sides, and were positively correlated with the degree of stenosis in the carotid artery (p<0.01). Moreover, the mean PI values on the asymptomatic side were positively associated with the mean PI values on the symptomatic side, in addition to the abovementioned parameters (p<0.01).

From the analyses performed in both the patient and the control group, we were unable to observe any significant relationship between the risk factors, CVR increases and PI values.

## DISCUSSION

Assessments of CVR by means of TCD and provocative vasodilatory tests are among the most commonly used tests for evaluating cerebral hemodynamic status in patients with carotid artery disease. Although several methods such as breath-holding, hyperventilation, CO_2_ challenge and the acetazolamide provocation test^17^ are widely used, we preferred to use active voluntary repetitive motor activity for the provocation test modality because of the evidence that such activity induces CVR improvements among stroke survivors^18^.

The mechanism postulated for this active motor movement of the fingers is associated with neuronal activation, dilatation of cerebral arterioles and increased regional cerebral blood flow caused by increased metabolism of the contralateral primary sensory-motor cortex, with the maximum increase of blood flow velocity on the posterior margin of the central sulcus^19^. Although TCD findings cannot provide any information about the mechanism of activation or the exact localization of changes in cerebral activity after these motor movements, they are widely used to assess cerebral autoregulation and collateral circulation.

Previous studies demonstrated that patients showed an association between impaired CVR and carotid occlusion^3,20,21^. The recent findings are also supported by the results from fMRI and TCD, which display slowed and reduced cortical hemodynamic responses to the different stimuli in the hemisphere ipsilateral to carotid stenosis^22^. Despite the absence of asymptomatic side difference, we demonstrated that the symptomatic side showed decreased CVR in all patients, in relation to the healthy subjects. However, there were some modifications to the methodology of our study, regarding the evaluation of PI values, such that the asymptomatic side was analyzed and patients were grouped according to the degree of stenosis.

King et al.^4^ described an association between CVR and the number of embolic signals in patients with asymptomatic carotid stenosis. This interesting result, which suggests that an interaction between hypoperfusion and embolism exists, can also guide us to an explanation for our findings about increased CVR and decreased PI values in group 1, compared with groups 2 and 3. Briefly, our study showed that both PI and CVR were impaired in patients with moderate to severe carotid artery stenosis.

Senescence causes atherosclerosis, decreased neuronal plasticity and greater rigidity of the vessel system, thus giving rise to constant vascular diameters. Therefore, patients of a given age might respond differently and show impaired reaction patterns to vasoactive stimuli. Schreiber et al.^23^ proved that there was no change in the diameter of the MCA after acetazolamide provocation testing, seen through high-resolution MRI on patients with occlusive extracranial carotid artery disease. In our study, the positive impact of age on the PI values and negative impact of age on the CVR was clearly observed. Indeed, the adverse interaction between CVR and PI entirely reflected the accuracy of this information.

Regarding the positive association of PI and CVR values on the symptomatic and the asymptomatic sides, we speculate that the parameters causing changes to cerebral autoregulation actually affect the entire brain, regardless of the side. However, better understanding of this association and the changes in the cerebral autoregulation is needed.

Lastly, some limitations to this study should be noted. These include the small number of patients, the lack of a standardized examination protocol for motor task administration due to absence of a device and the lack of any evaluation of compensatory mechanisms, including collateral blood flow through the ophthalmic artery, anterior communicating artery and posterior communicating artery. Nonetheless, despite these limitations, we believe that our study provides new insights.

In conclusion, the present study demonstrated that cerebral autoregulation and hemodynamic mechanisms are impaired in patients with carotid artery stenosis. Furthermore, with increasing degrees of stenosis, impairment of PI and CVR tends to become worse. Assessment of these two hemodynamic parameters in clinical practice might be helpful for monitoring the progress of carotid artery stenosis.
